# The Neglected Older Adults in Hereditary Angioedema: Insights From the ITACA Registry

**DOI:** 10.1002/clt2.70171

**Published:** 2026-05-21

**Authors:** Francesca Perego, Azzurra Cesoni Marcelli, Riccardo Senter, Lorenza Chiara Zingale, Antonio Gidaro, Valentina Popescu Janu, Francesco Arcoleo, Pietro Andrea Accardo, Mariangela Lo Pizzo, Andrea Zanichelli, Francesco Giardino, Andrea Caruso, Simone Giosuè Longhitano, Edoardo Cataudella, Alessandra Vultaggio, Andrea Matucci, Angelica Petraroli, Roberta Gatti, Giuseppe Spadaro, Luisa Brussino, Stefania Nicola, Luca Lo Sardo, Maria Domenica Guarino, Federica Ruin, Luca Ranucci, Donatella Bignardi, Marica Giliberti, Paolo Borrelli, Paola Triggianese, Massimo Triggiani, Caterina Colangelo, Paola Lucia Minciullo, Tiziana Maria Angela De Pasquale, Chiara Beatrice Cogliati, Giada De Angeli, Davide Firinu, Vincenzo Montinaro, Mauro Cancian

**Affiliations:** ^1^ Department of Internal Medicine Istituti Clinici Scientifici Maugeri IRCCS Milan Italy; ^2^ Department of Systems Medicine University Hospital of Padua Padua Italy; ^3^ Department of Biomedical and Clinical Sciences L. Sacco Hospital, University of Milan Milan Italy; ^4^ Internal Medicine L. Sacco Hospital, ASST‐fbf‐sacco Milan Italy; ^5^ Clinical Pathology and Immunology Azienda Ospedaliera Ospedali Riuniti Villa Sofia‐Cervello Palermo Italy; ^6^ Department of Biomedical Sciences for Health University of Milan Milan Italy; ^7^ Operative Unit of Medicine, Angioedema Center IRCCS Policlinico San Donato Milan Italy; ^8^ A.O.U. Policlinico “G.Rodolico‐San Marco” Catania Italy; ^9^ Department of Clinical and Experimental Medicine University of Florence Florence Italy; ^10^ Immunoallergology Unit Careggi University Hospital Florence Italy; ^11^ Internal Medicine and Immunology University of Naples Federico II Naples Italy; ^12^ Department of Translational Medical Sciences University of Naples Federico II Naples Italy; ^13^ Allergy and Clinical Immunology Unit, Department of Medical Sciences University of Torino & Mauriziano Hospital Torino Italy; ^14^ Allergy Unit Hospital of Civitanova Marche Civitanova Marche Italy; ^15^ San Martino Hospital Genova Italy; ^16^ Policlinico di Bari Bari Italy; ^17^ Dermatology and Allergology Unit Ospedale Beauregard Aosta Italy; ^18^ Department of Biomedicine and Prevention Tor Vergata University Rome Italy; ^19^ University of Salerno Salerno Italy; ^20^ Allergology Out‐Patients Unit Pescara Italy; ^21^ Allergy and Clinical Immunology Unit, Department of Clinical and Experimental Medicine University of Messina Messina Italy; ^22^ Azienda Ospedaliero—Universitaria Maggiore della Carità of Novara Novara Italy; ^23^ Clinical Research Service IRCCS Policlinico San Donato San Donato Milanese Italy; ^24^ Department of Medical Sciences and Public Health University of Cagliari Cagliari Italy; ^25^ LUM University “Giuseppe Degennaro”, Casamassima (BA), and “Miulli” General Hospital Acquaviva delle Fonti (BA) Italy

**Keywords:** hereditary angioedema, ITACA, long‐term prophylaxis, older adults, registry

## Abstract

**Background:**

Hereditary Angioedema due to C1‐inhibitor deficiency (HAE‐C1INH) is a rare disease that affects individuals of all ages, however, older adults have never been characterized in terms of disease severity, comorbidities, and treatments. The aim was to identify the clinical characteristics of and therapeutic approaches in HAE‐C1INH patients aged 65 and older.

**Methods:**

Data from the ITACA (Italian network for Hereditary and Acquired Angioedema) Registry were prospectively collected for 10‐month.

**Results:**

Data from 647 HAE‐C1INH, patients including 343 females (53%), were collected: 114 patients (17.6%) were aged 65 and older (68 females; 58.6%). Group mean age was 74.3 ± 7.5 years, mean age at first‐symptom‐onset was 19 ± 15 years, and mean age at diagnosis was 45.6 ± 14.3 years. Common comorbidities were: hypertension (59.6%), dyslipidaemia (28.9%), coronary artery disease (14.0%), diabetes (14.0%), endocrinopathies (14.0%), neoplasia (12.3%) and B/C hepatitis (11.4%). Half of the older patients (49%) experienced at least one attack during the study period, and 8 patients (7%) had an attack frequency of > 0.5 attacks/month. Long‐term prophylaxis (LTP) was the treatment of choice in 45 patients (39.5%) and represented 15.9% of overall LTP prescriptions: 60% were treated with lanadelumab, 22.2% with attenuated androgens, 13.3% with berotralstat, and 4.5% with sub‐cutaneous C1INH concentrate. Gender differences were not detected in any of the variables analyzed.

**Conclusion:**

Older patients with HAE‐C1INH constitute a relevant subgroup, characterized by persistent disease activity and comorbidities. The availability of new therapies and guideline recommendations are driving an increase in LTP use, although shifting from older non‐specific treatments, especially androgens, is still incomplete.

**Abbreviations**
AA: attenuated androgens
AE‐QoL: angioedema quality of life
BK: bradykinin
C1INH: C1‐inhibitor
eCRF: electronic case report form
GDPR: general data protection regulation
HAE: hereditary angioedema
HAE‐C1INH: hereditary angioedema due to C1‐inhibitor deficiency
ITACA: Italian network for hereditary and acquired angioedema
LTP: long‐term prophylaxis
ODT: on‐demand treatment
pd: plasma derived
sc: subcutaneous

## 1 Introduction

Hereditary Angioedema (HAE) due to C1‐inhibitor (C1INH) deficiency (HAE‐C1INH) is a rare genetic disorder characterized by recurrent attacks of subcutaneous or submucosal swelling that usually affect the face, upper airway, extremities, gastrointestinal tract, or genitalia [1, 2, 3].

The disease is more frequently caused by mutations in the SERPING1 gene, and is transmitted as an autosomal dominant trait, but in 15%–20% of the cases a *de novo* mutation can occur [4]. The defect of the C1INH protein, which is the main protease of bradykinin (BK), can be either quantitative (HAE‐C1INH Type 1) or qualitative (HAE‐C1INH Type 2): the overproduction of BK, related to the lack of activity of C1INH, increases endothelial permeability and elicits angioedema attacks [5]. Recurrent swelling causes significant morbidity and can be life threatening, especially when the swelling affects the airway [6]. Onset of symptoms typically occurs within the first 2 decades of life (often by age 10–11 years), with intensity of attacks worsening during puberty [7, 8]. However, HAE‐C1INH attacks may occur at any age and recur throughout a patient's lifetime [6].

Although survival in patients with HAE‐C1INH has improved over recent years, mainly thanks to the expanding range of diagnostic tools and therapeutic options [9, 10], HAE management still represents a clinical challenge [11]. Moreover, over the past 50 years, the aging of the population in developed countries is accelerating, and estimates for 2050, show that the proportion of people aged ≥ 65 will reach 36% of the total population [12]. However, older HAE patients have never been fully characterized in terms of disease severity, comorbidities, or attack management, and are underrepresented in clinical trials [13, 14]. To date, publications focused on HAE in older patients are scarce, generally related to the treatment of acute attacks with icatibant [14] or C1‐inhibitor concentrate [13]. The efficacy of long‐term prophylaxis (LTP) was tested in a subgroup analysis in clinical trial [15], and in real life [16], but generally in limited samples or sometimes described in single clinical cases [17]. Age‐related pharmacokinetic and pharmacodynamic changes, combined with the high prevalence of comorbidities requiring polypharmacy, highlight the critical need for careful monitoring of medication safety in this age group [18, 19].

Current consensus guidelines do not address issues specific to older adults [1, 5], and there remains a gap in research concerning the management in this age group, including general treatment strategies and definition of clinical outcomes. Moreover, the burden of disease in older patients with HAE‐C1INH presents unique challenges, including increased need for external support, reduced access to healthcare services, progressive decline in overall health status [20], and age‐related physiological changes such as menopause.

The older population of patients affected by HAE‐C1INH can in fact be regarded as a *special population*, which remains largely “invisible” to date despite its high and increasing prevalence. The aim of this study was to provide a comprehensive characterization of this specific patient group across the entire Italian territory. Specifically, we aimed to describe the clinical characteristics and ongoing real‐life treatments of HAE‐C1INH patients aged 65 and older, based on data from a large prospective cohort within the ITACA (Italian Network for Hereditary and Acquired Angioedema) Centers.

The primary outcome was the percentage of HAE‐C1INH patients aged 65 and older.

Secondary outcomes included:

- The number of the attacks during the observation period;
- The characteristics of attacks in terms of severity, localization, duration, acute treatment
- The number and percentage of comorbidities;
- The number and percentage of patients receiving LTP;
- The distribution of LTP prescriptions;
- The score of the Angioedema Quality of Life Questionnaire (AE‐QoL).

## 2 Methods

**FIGURE 1:**
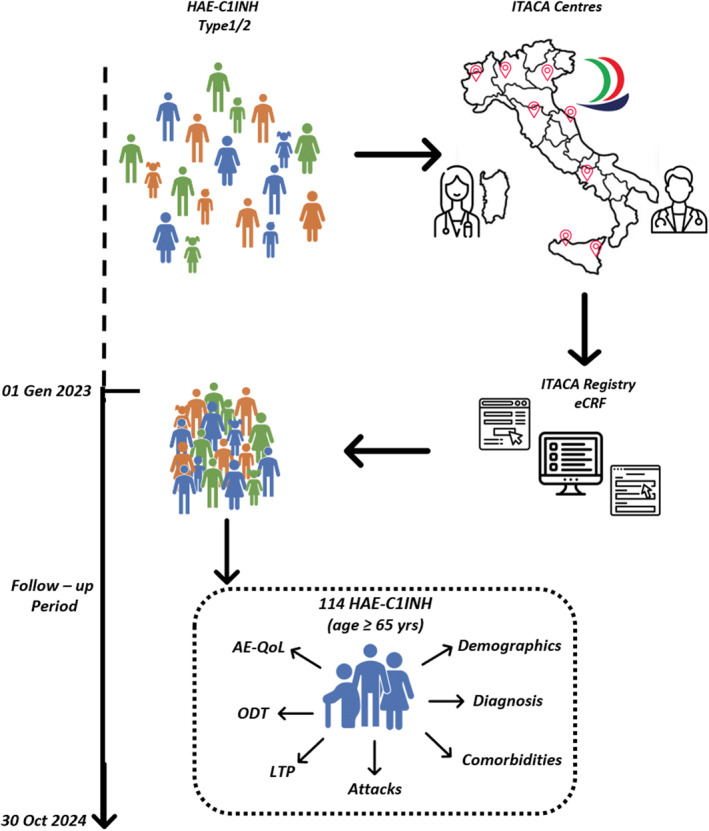
Study protocol. AE‐QoL, angioedema quality of life; eCRF, electronic Case Report Form; HAE‐C1INH 1/2, C1 Inhibitor Hereditary Angioedema Type 1 and 2; LTP, long‐term prophylaxis; ODT, on‐demand therapy; yrs, years.

This multicentric observational prospective study enrolled patients with a confirmed diagnosis of HAE‐C1INH Type 1/2 who provided informed consent to be included in the ITACA Registry (ClinicalTrials.gov ID NCT03828279). The registry was approved by the Ethical Committee (date 08 May 2017, approval number 11846) and adhered to Good Clinical Practice, and the Declaration of Helsinki. The study design is shown in Figure 1. It was conducted in 10 Italian reference centers of the ITACA network. After the inclusion the patients were followed‐up from January 1, 2023, to October 30, 2024. The diagnosis of HAE‐C1INH was defined according to guidelines [1]: (1) a positive family history (although this may not be present in up to 25% of patients), (2) onset of symptoms in childhood/adolescence, (3) recurrent and painful abdominal symptoms, (4) occurrence of upper airway edema, (5) failure to respond to antihistamines, glucocorticoids, or epinephrine, (6) low antigenic and functional C1INH levels (Type 1); low functional C1INH levels (Type 2).

Data about demography, diagnosis, comorbidities, HAE attacks, on‐demand treatment (ODT), LTP treatment, were collected by electronic Case Report Form (eCRF). Patients were asked to enter the AngioEdema Quality of Life Questionnaire [21] (AE‐QoL) during the study period. AE‐QoL is a 17‐item scale, that measures disease‐specific impairment across four domains (Functioning, Fatigue/Mood, Fears/Shame, and Food) designed to evaluate the quality of life impairment due to angioedema attacks. It is scored as a percentage on a scale of 0–100, with lower scores indicating less impairment and better quality of life. We defined older patients as those aged ≥ 65 years, adhering to the standard definition set by the International Council for Harmonization (ICH) Harmonized Guidelines and used in most developed countries for geriatric population studies [22]. The characteristics of the attacks were inserted directly by the patients using a Web form and flowed into a staging area for physician validation, before being considered valid for statistics. Both patients and the physician were permitted to report the frequency of episodes occurring during the study period in cases where detailed documentation was lacking, and only number of attacks was available. One designated physician of each ITACA Reference Center downloaded pseudo‐anonymized data of patients followed by that Center.

The data processor (Burning Flame S.r.l. Milan, Italy) managed all data according to a specific contract and in compliance with current regulation on sensitive data security and processing. The Registry was designed following General Data Protection Regulation (GDPR) guidelines, and issues regular software and infrastructure enhancements as a part of the normal operational mode. The platform has configurable functionalities to support data quality management. It provides data format validation, integration to external qualified libraries, alerts, dashboards, advanced filters and queries, data change log. The registry quality control system periodically checks Registry entries and compliance of eCRF with the source data. For each information, the system grants traceability of time and author.

### 2.1 Statistical Analysis

Descriptive statistics for primary and secondary outcomes were provided according to the type of variable summarized: for quantitative variables standard quantitative statistics was performed (*N*, mean, standard deviation); for qualitative variables: frequency distribution (*N*) and percentages (%). Unpaired Student t‐test, or *χ*2 test were performed between female and male to compare continuous or categorical variable respectively. Differences in disease attack incidence between female and male were assessed using Poisson regression, assuming equal follow‐up time across individuals. Sex was included as a binary covariate, and incidence rate ratios (IRRs) with 95% confidence intervals (CIs) were estimated. Sensitivity analyses were performed by excluding individuals with a high number of disease attacks (> 12) and by restricting the analysis to participants with fewer than 10 attacks, to assess the robustness of sex‐related differences in incidence rates. Statistical analysis was performed using a commercial statistical software (Sigmaplot, Systat Software, Chicago IL, version 11.0).

## 3 Results

Data from 647 patients with HAE‐C1INH Type 1/2, 343 females (53%), were collected in the Registry from 10 Reference Centers. Of these, 114 patients, accounting for 17.6% of the total HAE‐C1INH population, were aged 65 and older (68 females, 58.6%; 95 Type 1, 83.3%), and were included in the study (Table 1). All the patients were White. The mean age was 74.3 ± 7.5 years, the mean age at first symptom onset was 19 ± 15.5 years, and the mean age at diagnosis was 45.6 ± 14.3 years. Forty‐four patients (38.6%) had suffered laryngeal attacks in the past.

**TABLE 1:** Characteristics of the population aged 65 and over.

	Female	Male	Total
Number (%)	68 (58.6)	46 (40.4)	114 (100.0)
Age, yrs, mean (SD)	74.9 (8.4)	73.2 (5.8)	74.3 (7.5)
Age, yrs, (min‐max)	65–94	65–90	65–94
Type 1, *n* (% total)	56 (82.3)	39 (84.8)	95 (83.3)
Type 2, *n* (% total)	12 (17.6)	7 (15.2)	19 (16.7)
Age first symptoms, yrs, mean (SD)	16.9 (11.5)	21.5 (18.9)	19 (15.5)
Age at diagnosis, yrs, mean (SD)	45.4 (13.6)	45.9 (15.2)	45.6 (14.3)
C4, % of normal value (SD)	24.2 (16.9)	18.6 (16.8)	21.9 (17.1)
C1 INH, % of normal value (SD)	34.9 (42.3)	25.4 (16.6)	30.9 (34.2)
C1 INH functional, % of normal value (SD)	17.2 (12.4)	20.9 (12.1)	18.7 (12.4)
Laryngeal attacks, *n* (%)	26 (38.2)	18 (39.1)	44 (38.6)
Total number of attacks (*n*)	300	87^a^	387
Patients with attacks, *n* (%)	34 (50)	22 (47.8)	56 (49)
Patient with attack rate > 0.5 (attacks/months), *n* (%)	6 (8.8)	2 (4.3)	8 (7)

^a^ **=** IRR = 2.33, 95% CI 1.84–2.96; *p* < 0.001 Male versus Female.

Differences in the incidence of attacks between females and males were not observed. Indeed, in a sensitivity analysis restricted to individuals with fewer than 10 attacks, no meaningful difference in incidence rates between females and males was detected, with incidence rate ratios close to unity.

**FIGURE 2:**
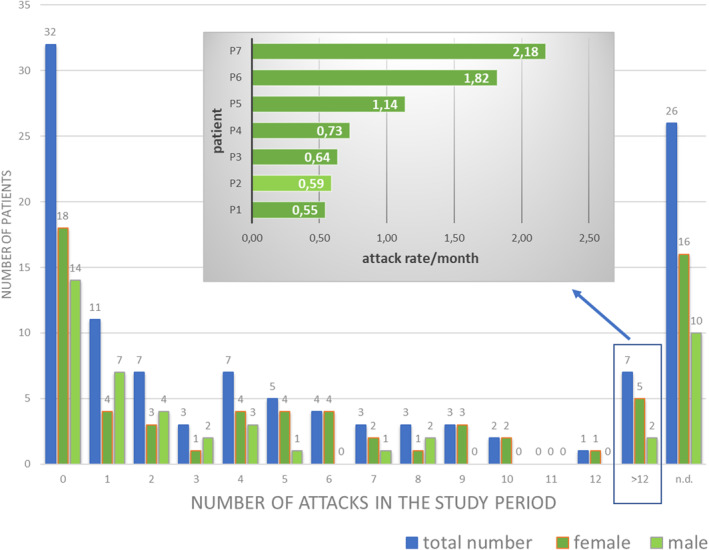
Distribution of the number of attacks in HAE‐C1INH aged 65 and older. Gray‐box reports the detail of the attack rate per month of the 7 patients who experienced more than 12 attack during the study period. n.d., not detected.

However, as five females and two males experienced more than 12 attacks during follow‐up, considering the data altogether, the females had a higher incidence of disease attacks (IRR = 2.33, 95% CI 1.84–2.96; *p* < 0.001). This result was largely attributable to a small subset of patients with a high attack burden (Figure 2).

Differences between female and male were not observed (*p* > 0.05) for all the other variables.

All the patients were followed up for the duration of the study and no deaths occurred.

Of the 114 HAE‐C1INH patients included, the exact number of attacks occurring during the study period was known in 88 patients (77.2%) (Figure 2), 32 patients did not experience any attacks, while 56 reported at least one attack; among these, 22 patients reported attacks without providing details, whereas 34 provided attack characteristics, including 26 who consistently reported complete information and 8 who did so inconsistently.

A total of 387 attacks were registered (Table 1, Figure 2). Around half of the older patients (49%) experienced at least one attack, with 8 patients (7%) showing an attack frequency of > 0.5 attacks per month (Table 1, Figure 2). Differences between male and female were not observed.

For 145 (34%) attacks details were provided by 34 patients (Table 2): 20.7% attacks were severe, the main localization was abdominal (53.8%), the mean time to resolution was 32.7 h, 44.8% occurred in patients without any kind of prophylaxis. Among the 34 patients without prophylaxis, seven experienced one attack and four experienced two attacks, whereas among patients receiving prophylaxis, five experienced one attack and one experienced two attacks. Eight patients without prophylaxis and nine patients receiving prophylaxis experienced three or more attacks.

**TABLE 2:** Attack characteristics in HAE‐C1INH aged 65 and older.

Detailed attacks, *n*	145
Severity	
Mild, *n* (%)	46 (31.72)
Moderate, *n* (%)	63 (43.45)
Severe, *n* (%)	30 (20.69)
Not reported, *n* (%)	6 (4.14)
Localization	
Abdomen, *n* (%)	78 (53.79)
Cutaneous, *n* (%)	56 (38.62)
Throat/oral cavity, *n* (%)	4 (2.76)
Abdomen and cutaneous^a^, *n* (%)	4 (2.76)
Not reported, *n* (%)	3 (2.07)
Time to resolution, hours, mean (SD)	32.7 (28.5)
Number of acute treatments	
1, *n* (%)	80 (55.17)
2, *n* (%)	16 (11.03)
> 2, *n* (%)	9 (6.20)
Not treated, *n* (%)	23 (15.80)
Not detected, *n* (%)	17 (11.80)
Ongoing prophylaxis	
None, *n* (%)	65 (44.83)
Lanadelumab, *n* (%)	31 (21.38)
Berotralstat, *n* (%)	16 (11.03)
Attenuated androgens, *n* (%)	13 (8.97)
IV C1‐INH, *n* (%)	5 (3.45)
sc C1‐INH, *n* (%)	5 (3.45)
Other, *n* (%)	10 (6.89)

Abbreviations: IV C1‐INH, intravenous C1 inhibitor; sc C1‐INH, subcutaneous C1 inhibitor.

^a^ Attacks simultaneously involving the abdomen and the skin.

Comorbidities collected at the beginning of the study are summarized in Table 3. The most represented comorbidities were hypertension (59.6%), dyslipidaemia (28.9%), coronary artery disease (14.0%), diabetes (14.0%), endocrinopathies (14.0%), neoplasia (12.3%), and B/C hepatitis (11.4%). The distribution of comorbidities was similar in males and females (*p* > 0.05 for all the variables). The incidence of comorbidities during the period of observation was not collected.

**TABLE 3:** Comorbidities in HAE‐C1INH aged 65 and over.

	Female *N* = 68	Male *N* = 46	Total *N* = 114
Hypertension, *n* (%)	39 (57.3)	29 (63.0)	68 (59.6)
Dyslipidemia, *n* (%)	20 (29.4)	13 (28.3)	33 (28.9)
Coronary artery disease, *n* (%)	6 (8.8)	10 (21.7)	16 (14.0)
Endocrinopathies, *n* (%)	12 (17.6)	4 (8.7)	16 (14.0)
Diabetes, *n* (%)	8 (11.8)	8 (17.4)	16 (14.0)
Neoplasia, *n* (%)	8 (11.8)	6 (13.0)	14(12.3)
Appendectomy, *n* (%)	11 (16.2)	3 (6.5)	14 (12.3)
B And C hepatitis, *n* (%)	6 (8.8)	4 (8.7)	10 (11.4)
Gastroesophageal reflux disease GERD/gastritis, *n* (%)	7 (10.3)	2 (4.3)	9 (7.9)
Osteoporosis, *n* (%)	8 (11.8)	1 (2.2)	9 (7.9)
Prostatic hypertrophy, *n* (%)	n.a.	8 (17.4)	8 (7.0)
Deep venous thrombosis, *n* (%)	3 (4.4)	3 (6.5)	6 (5.3)
Atrial fibrillation/flutter, *n* (%)	5 (7.3)	1 (2.2)	6 (5.3)
Asthma/COPD, *n* (%)	4 (5.9)	2 (4.3)	6 (5.3)
Stroke/transient ischemic attack, *n* (%)	4 (5.9)	0	4 (3.5)
Anxiety/depression, *n* (%)	3 (4.4)	1 (2.2)	4 (3.5)
Cerebral hemorrhage, *n* (%)	1 (1.5)	2 (4.3)	3 (2.6)
Other arrythmias, *n* (%)	2 (2.9)	1 (2.2)	3 (2.6)
Chronic kidney disease, *n* (%)	2 (2.9)	1 (2.2)	3 (2.6)
Pulmonary embolism, *n* (%)	2 (2.9)	0	2 (1.7)
Acute pancreatitis, *n* (%)	1 (1.5)	1 (2.2)	2 (1.7)

*Note:* Percentages refers to column percentage.

Abbreviation: n.a., not applicable.

All the patients had ODT available for the treatment of the attacks: intravenous plasma derived (pd) C1INH concentrate 20 IU/kg was prescribed in 32 patients, subcutaneous (sc) icatibant 30 mg in 48 patients, and 12 had access to both medications. Ten patients had not reported ODT regimen in the registry.

During the study period, older patients on LTP represented 15.9% of all LTP prescriptions (data not shown in table), with LTP ongoing in 45 patients (39.5%) (Table 4). Among these, 27 patients (60.0%) were treated with sc lanadelumab 300 mg once or twice per month, 10 (22.2%) with oral attenuated androgens (AA) at various dosage not exceeding 200 mg daily, 6 (13.3%) with oral berotralstat, and 2 (4.5%) with sc pdC1INH concentrate 60 IU/kg twice every 3–4 days (Table 4).

**TABLE 4:** Long‐term prophylaxis in HAE‐C1INH aged 65 and older.

	Female *N* = 68	Male *N* = 46	Total *N* = 114
LTP, *n* (%)	25 (36.8)	18 (39.1)	45 (39.5)
Lanadelumab, *n* (%)	16 (23.5)	11 (23.9)	27 (60.0)^a^
Berotralstat, *n* (%)	3 (4.4)	3 (6.5)	6 (13.3)^a^
Attenuated androgens, *n* (%)	6 (8.8)	4 (8.7)	10 (22.2)^a^
sc C1‐INH, *n* (%)	2 (2.9)	0	2 (4.4)^a^

Abbreviations: LTP, Long‐Term Prophylaxis; sc C1‐INH, subcutaneous C1 inhibitor.

^a^ Percentages refers to column percentage of patients on LTP.

All the patients treated with lanadelumab, berotralstat, and sc pdC1INH switched from AA LTP prior or during the period of observation, while 4 patients on AA had discontinued the therapy without replacing with alternative medications.

AE‐QoL were completed by 25 patients (21.9%) who filled 36 questionnaires during the duration of the study (Appendix 1). The total score of the AE‐QoL was 34 ± 12.2 points, without differences between genders in the total score and in the score of the four domains (Appendix 1).

## 4 Discussion

### 4.1 Population

This study includes the largest, prospectively observed, cohort of HAE‐C1INH patients aged 65 years and older reported so far. Our national survey shows that approximately one fifth of all HAE‐C1INH patients in Italy belong to this age group, representing a substantial proportion that physicians and healthcare providers must be aware of. Elderly patients with HAE‐C1INH have been historically underrepresented or excluded from pivotal clinical trials, limiting the generalizability of trial‐based evidence to this population. In this context, real‐world data from the ITACA study provide important complementary insights, helping to bridge existing knowledge gaps and better inform clinical practice.

According to the National Institute of Statistics (ISTAT; www.istat.it), updated as of March 2025, the proportion of the Italian population aged 65 and older is 24.7%. The prevalence of HAE‐C1INH among patients in this age group appears to be of similar magnitude, also taking into account the likely presence of undiagnosed cases.

In the population here described, the age at diagnosis was 45 years, with a diagnostic delay of approximately 25 years. This result is likely due to the fact that the population in the study was diagnosed several years ago, when knowledge of the disease and availability of diagnostic tests were poor. Recent findings, demonstrated a decrease in the delay in diagnosis due to increased physician awareness of this rare condition and advances in diagnostic tools [23].

### 4.2 Attacks

Nearly half of the older patients still have attacks and 7% of them have an attack rate consistent with a scarce control of symptoms. Although detailed information was available for only 34% of all attacks, this represents the largest report on attack characteristics in this age group and highlights the substantial disease burden: 64% of attacks were moderate or severe, and 55% occurred despite prophylaxis. Consistently with the marked heterogeneity in disease activity observed in this cohort, the higher attack incidence among females was largely driven by the small subgroup of patients with a high attack burden. When analyses were restricted to individuals with fewer attacks, no relevant sex‐related differences in incidence rates were observed, suggesting that disease activity in older patients is characterized by substantial inter‐individual variability rather than by a homogeneous sex effect.

While a direct comparison with a younger control group was not the aim of this study, our findings can be contextualized against data from the general HAE‐C1INH population. Previous retrospective analyses, such as the IOS study [14], suggested that attack frequency does not significantly decline with age. Our prospective real‐life data confirm this observation: with nearly half of the cohort (49%) experiencing attacks and a subset showing high disease activity, we prove that HAE‐C1INH does not necessarily attenuate in the elder adult.

### 4.3 Comparison by Sex

No clear sex‐related differences were observed. All female patients were postmenopausal and were not receiving hormone replacement therapy. Since the study did not include retrospective information on the number of attacks prior to the 22‐month observation period, the contribution of hormonal changes to the occurrence of attacks could not be assessed. We did not observe more severe disease in females as a group, in contrast to what was previously reported in unselected HAE‐C1INH populations [24]. However, high disease‐burden patients in our population are mostly female. We speculate that hormonal differences do not significantly impact disease activity in older patients, unlike the comparison observed between women of childbearing age and men in the same age range [25]. Nonetheless some female patients could be more symptomatic for unclear reasons.

### 4.4 Comorbidities

The reported comorbidities provide a detailed characterization of the clinical profile of older HAE‐C1INH patients. Nevertheless, caution is warranted in attempting comparisons with the general population unaffected by angioedema, as data from a rare disease registry are collected using methodologies and for purposes that differ substantially from those of population‐based epidemiological surveys. Arterial hypertension may represent an exception, given its high prevalence in the general population and its consistent representation in our cohort [26], suggesting limited variability across settings.

PASSI d’Argento [27], an Italian nationwide surveillance program of individuals aged ≥ 65 years, estimated a hypertension prevalence of approximately 60%, which is comparable to that observed in our cohort. With regard to cardiovascular comorbidities, several recent studies including patients across all age groups have reported an increased burden of comorbid conditions among HAE‐C1INH patients [28, 29]. A Swedish population‐based study reported an increased risk of cardiovascular disease, as well as arterial and venous thromboembolic events, hypertension, and dyslipidemia in HAE‐C1INH patients [29]. However, among the 239 HAE‐C1INH patients included, only 57 were aged ≥ 60 years, and this age threshold differs from that applied in the present analysis. Moreover, the results were not stratified by age, precluding a direct comparison with our findings. Similarly, a single‐center Italian survey [28] reported a higher incidence of hypertension, hypercholesterolemia, diabetes mellitus, hepatic angioma, and focal nodular hyperplasia among patients treated with attenuated androgens. These medications may represent an additional risk factor for the occurrence of cardiovascular, metabolic or hepatic comorbidities [30]. In that study, comparisons of the prevalence of comorbidities with the general population were indirect and, again, not stratified by age, limiting the comparability with our data.

For less prevalent conditions, such as autoimmune disease, the comorbidity data presented here should just be regarded as descriptive and hypothesis‐generating.

No patients from our cohort reported allergies and few were affected from autoimmune disease. Data on the prevalence of allergic and autoimmune diseases in HAE‐C1INH patients are limited and heterogeneous particularly for older adults. A Canadian self‐reported survey [31] suggested a higher frequency of autoimmune diseases and allergies in HAE‐C1INH compared with the general population, although the study included very few individuals aged ≥ 65 years and relied on unverified self‐reported diagnoses. In contrast, a Swedish study [29] based on physician diagnoses and registry data found an increased overall risk of autoimmune diseases in HAE‐C1INH patients, but did not confirm a higher prevalence of allergic diseases, highlighting the uncertainty and methodological limitations of the available evidence. This discrepancy may be due to different methodological approaches, while our data on immune‐mediated disease do not allow to draw any meaningful conclusion.

From a clinical stand‐point, these patients appear to be complex, which compels patients, caregivers, and physicians to manage multidrug regimens and the progressive loss of self‐sufficiency, in addition to the challenges of angioedema management.

### 4.5 Long‐Term Prophylaxis

The proportion of patients on LTP here reported is 39%, with lanadelumab being the most common prescribed treatment for several reasons: first of all, this medication was the first treatment approved for LTP, and long‐term data on efficacy, safety, and costs are available [32]. Moreover, the longer and flexible dosing interval, together with the low volume required for subcutaneous administration, make lanadelumab more convenient to administer than iv or sc pdC1‐INH and reduce the need for caregiver assistance. With regard to berotralstat, which—like lanadelumab—inhibits plasma kallikrein, it should be noted that this oral drug became available in Italy only during the latter part of the study's observation period.

Worldwide, the percentage of patients on LTP is highly variable, mainly depending on drug availability. Indeed, data from the Brazilian Mulicenter Registry [33] reported that only 4.8% used first‐line options for LTP, such as lanadelumab or subcutaneous/intravenous pdC1INH, favoring attenuated androgens that were used in half of patients. In Italy, all the approved drugs for LTP are available, but first‐line option are preferred.

Our data add specific information concerning older patients and map out the journey on how the new drugs are replacing the older one. This transition is still ongoing, indeed 22% of LTP treated patients are still on AA. This could be partly explained by the fact that patients who are well controlled on low doses (e.g., 50 mg/day) are often reluctant to modify their treatment regimen. Furthermore, patients may underestimate adverse effects that remain clinically silent. In Italy, the use of AA in prophylaxis‐naïve patients is generally regarded as a last‐line therapeutic option.

### 4.6 Quality of Life

The evaluation of quality of life in the older population remains an open issue. Adherence to completing the AE‐QoL was low, and based on the available data, no gender differences were observed. However, this raises the question of whether the AE‐QoL is an adequate tool for assessing quality of life in older adults and whether greater medical attention to this aspect is needed in this population.

### 4.7 Limitations

Our study has several limitations. The study population included only White patients from a single country (Italy). While this ensures genetic and cultural homogeneity, it limits the generalizability of our results to other ethnic groups and to healthcare systems with different reimbursement policies for HAE‐C1INH medications. In addition, data were collected from specialized referral centers (ITACA network), which may introduce a selection bias toward patients with more severe disease or higher treatment adherence compared to the general unselected population. Moreover, the lack of retrospective data on disease activity prior to the observation period prevented a direct longitudinal assessment of symptom evolution over individual lifespans.

In the present article we chose to focus primarily on the population experiencing attacks, rather than attempting a simultaneous per‐patient and per‐attack analysis. A comprehensive analysis of the characteristics and management will be the subject of a separate publication. Indeed, incomplete documentation of attack characteristics represents a relevant limitation of this study and may introduce selection bias in the analysis of attack‐related features. Potential sources of bias in reporting attacks could include variable patient compliance, differences in ability to use digital reporting tools, and likely underreporting of mild attacks.

The completion rate of AE‐QoL in this report was low. The AE‐QoL has demonstrated methodological applicability in geriatric HAE‐C1INH populations, having been completed by a significant proportion of patients aged > 60 years in the original validation cohort [21]. However, the lack of validation of the Italian version of the AE‐QoL, and a limited competence with digital devices may have affected questionnaire comprehension and completion in our study population.

Finally, the study was not designed to make a precise estimate of the comorbidities that can be therefore underreported, especially if mild.

## 5 Conclusion

Older patients with HAE‐C1INH represent a clinically relevant subgroup, in whom the disease remains active despite advanced age and who frequently present with a high burden of comorbidities. Our findings provide novel insights into disease activity and treatment patterns, showing that long‐term prophylaxis is increasingly based on modern therapies, although the transition from older agents is still incomplete. These results emphasize the need for greater awareness and tailored management strategies for older individuals with HAE‐C1INH, an often‐neglected population within both clinical practice and research.

## Author Contributions

**Francesca Perego:** conceptualization, data Curation, formal Analyses, investigation, methodology, project administration, resources, software, visualization, writing – original draft. **Azzurra Cesoni Marcelli:** writing – review and editing, writing – original draft, conceptualization, resources. **Riccardo Senter:** writing – original draft, methodology, writing – review and editing, data curation, resources. **Lorenza Chiara Zingale:** writing – review and editing, resources. **Antonio Gidaro:** writing – review and editing, resources. **Valentina Popescu Janu:** writing – review and editing, resources. **Francesco Arcoleo:** writing – review and editing, resources. **Pietro Andrea Accardo:** writing – review and editing, resources. **Mariangela Lo Pizzo:** writing – review and editing, resources. **Andrea Zanichelli:** writing – review and editing, resources, methodology. **Francesco Giardino:** investigation, resources, writing – review and editing. **Andrea Caruso:** investigation, resources, writing – review and editing. **Simone Giosue Longhitano:** writing – review and editing, resources. **Edoardo Cataudella:** investigation, resources, writing – review and editing. **Alessandra Vultaggio:** writing – review and editing, resources. **Andrea Matucci:** investigation, resources, writing – review and editing. **Angelica Petraroli:** writing – review and editing, resources. **Roberta Gatti:** writing – review and editing, resources. **Giuseppe Spadaro:** writing – review and editing, resources. **Luisa Brussino:** writing – review and editing, resources. **Stefania Nicola:** writing – review and editing, resources. **Luca Lo Sardo:** writing – review and editing, resources. **Maria Domenica Guarino:** writing – review and editing, resources. **Federica Ruin:** writing – review and editing, methodology, data curation, software. **Luca Ranucci:** writing – review and editing, software, resources, visualization. **Donatella Bignardi:** writing – review and editing, resources. **Marica Giliberti:** writing – review and editing, resources. **Paolo Borrelli:** writing – review and editing, resources. **Paola Triggianese:** writing – review and editing, resources. **Massimo Triggiani:** writing – review and editing, resources. **Caterina Colangelo:** writing – review and editing, resources. **Paola Lucia Minciullo:** investigation, resources, writing – review and editing. **Tiziana Maria Angela De Pasquale:** writing – review and editing, resources. **Chiara Beatrice Cogliati:** writing – review and editing, resources. **Giada De Angeli:** data curation, resources, writing – review and editing. **Davide Firinu:** writing – review and editing, resources. **Vincenzo Montinaro:** writing – review and editing, resources. **Mauro Cancian:** conceptualization, project administration, resources, writing – review and editing, validation, supervision, funding acquisition.

## Funding

The authors have nothing to report.

## Conflicts of Interest

F.P.: support and/or speaker/consultancy fees or travel grant from BioCryst, CSL Behring, Takeda, Otsuka, and Pharvaris; A.C.M.: grant from CSL Behring, Takeda, and BioCryst; clinical trial investigator for BioCryst, and Takeda; R.S.: speaker/consultancy fees from BioCryst, Takeda, Kalvista, Blueprint. CSL Behring, Alk Abello, Anallergo, and Novartis; L.C.Z.: travel grant from CSL Behring, Takeda, and BioCryst; clinical trial investigator for BioCryst, and Takeda; A.G.: speaker/consultancy fees from BioCryst Pharmaceuticals, CSL Behring, and Takeda; V.P.: no conflict of interest; F.A.: research support and/or speaker/consultancy fees from CSL Behring and Takeda; P.A.A.: speaker/consultancy fees from BioCryst, KalVista,Takeda, and CSL Behring; M.L.P.: no conflict of interest; A.Z.: support from BioCryst, KalVista, and Pharvaris; consulting fees from BioCryst, CSL Behring, KalVista, Takeda; honoraria from Astria, BioCryst, CSL Behring, Intellia, KalVista, Pharvaris, PINT Pharma, and Takeda; advisory boards for Astria, BioCryst, CSL Behring, Intellia, KalVista, Pharvaris, and Takeda; F.G.: speaker/consultancy fees or travel grant from BioCryst, CSL Behring, KalVista Pharmaceuticals and Takeda; A.C.: no conflict of interest; S.G.L.: no conflict of interest; E.C.: no conflict of interest; A.V.: speaker fees and funding to attend conferences from BioCryst; A.M.: speaker/consultancy fees or travel grant from BioCryst, CSL Behring, and Takeda; A.P.: consultancy fees or travel grant from BioCryst, CSL Behring, KalVista Pharmaceuticals and Takeda; R.G.: no conflict of interest; G.S.: grant research support/consultancy fees from CSL Behring, Takeda, and BioCryst; conference attendance or travel support from CSL Behring, Takeda, and BioCryst; participation on advisory boards for CSL Behring, Takeda, and BioCryst; L.B.: consultancy fees or travel grant from BioCryst,Takeda, and CSL Behring; S.N.: no conflict of interest; L.L.S.: no conflict of interest; M.D.G.: speaker fees or travel grant from Takeda, Biocryst, CSL Behring; F.R.: no conflict of interest; D.B.: speaker for BioCryst; M.G.: speaker fees or travel grant from BioCryst, Kyowa Kirin; BioCryst, Amicus, Kyowa Kirin, CSL Behring, Sanofi, Takeda, and AstraZeneca; P.B.: no conflict of interest; P.T.: speaker fees or travel grant from from BioCryst, CSL Behring, Takeda, and KalVista, Otsuka and Jannsen; M.T.: received fees for advisory boards from CSL Behring, Takeda, and Biocryst.; C.C.: no conflict of interest; P.L.M.: no conflict of interest; T.M.A.D.P.: received travel grant by CSL Bering, Biocryst, Sanofi, AstraZeneca, Menarini. Received consultation fee by Sanofi; C.B.C.: no conflict of interest; G.D.A.: no conflict of interest; D.F.: speaker for Takeda, CSL Behring, Novartis, GSK; V.M.: speaker/consultancy fees from BioCryst, CSL Behring, Takeda; M.C.: grant research support/speaker/consultancy fees from BioCryst, Chiesi, CSL Behring, Kalvista, Novartis, Otsuk, Pharming, Pharvaris, Sanofi and Takeda

## Data Availability

The data that support the findings of this study are available from the corresponding author upon reasonable request.

## Appendix 1

Table A1

**TABLE A1:** Angioedema quality of life questionnaire.

	Female *N* = 68	Male *N* = 46	Total *N* = 114
Number of patients who filled AE‐QoL, *n* (%)	19 (27.9)	6 (13.0)	25 (21.9)
Number of AE‐QoL completed	27	9	36
Total score, points, mean (SD)	36.1 (11.7)	27.7 (12.1)	34.0 (12.2)
Functioning, points, mean (SD)	7.3 (3.6)	6.9 (3.6)	7.2 (3.5)
Fatigue/mood, points, mean (SD)	11.1 (4.3)	8.6 (3.6)	10.5 (4.3)
Fear/shame, points, mean (SD)	13.9 (5.2)	8.6 (3.6)	12.6 (5.3)
Food, points, mean (SD)	3.9 (2.1)	3.7 (2.4)	3.5 (2.2)

Abbreviations: AE‐QoL, Angioedema Quality of Life Questionnaire; SD, Standard Deviation.
